# Differentiation of meat species of raw and processed meat based on polar metabolites using ^1^H NMR spectroscopy combined with multivariate data analysis

**DOI:** 10.3389/fnut.2022.985797

**Published:** 2022-09-30

**Authors:** Christina Decker, Reiner Krapf, Thomas Kuballa, Mirko Bunzel

**Affiliations:** ^1^Karlsruhe Institute of Technology (KIT), Department of Food Chemistry and Phytochemistry, Karlsruhe, Germany; ^2^Chemisches und Veterinäruntersuchungsamt Karlsruhe, Karlsruhe, Germany; ^3^Bosch Power Tools, Leinfelden-Echterdingen, Germany

**Keywords:** ^1^H NMR spectroscopy, meat, authentication, species, multivariate statistical analysis

## Abstract

Meat species of raw meat and processed meat products were investigated by ^1^H NMR spectroscopy with subsequent multivariate data analysis. Sample preparation was based on aqueous extraction combined with ultrafiltration in order to reduce macromolecular components in the extracts. ^1^H NMR data was analyzed by using a non—targeted approach followed by principal component analysis (PCA), linear discrimination analysis (LDA), and cross-validation (CV) embedded in a Monte Carlo (MC) resampling approach. A total of 379 raw meat samples (pork, beef, poultry, and lamb) and 81 processed meat samples (pork, beef, poultry) were collected between the years 2018 and 2021. A 99% correct prediction rate was achieved if the raw meat samples were classified according to meat species. Predicting processed meat products was slightly less successful (93 %) with this approach. Furthermore, identification of spectral regions that are relevant for the classification *via* polar chemical markers was performed. Finally, data on polar metabolites were fused with previously published ^1^H NMR data on non-polar metabolites in order to build a broader classification model and to improve prediction accuracy.

## Introduction

The terms “food fraud” or “food adulteration” gained unfortunate popularity in recent years, particularly as a result of the horse meat scandal in 2013 (1, 2). Food fraud generally refers to the act of placing food on the market with characteristics that do not match those advertised. This may be done intentionally in order to achieve an economic advantage and to maximize profits by lowering production costs. Areas of food adulteration are versatile, examples of this are false declarations of origin, husbandry, and cultivation information, the addition of undeclared ingredients, and non-compliance with quality and purity specifications (3).

In the area of meat fraud, this applies to changing the composition of meat and meat products, for example by adding water and/or undeclared additives. Other possible areas of fraud in the meat sector include incorrect information about the geographical origin and whether the meat was organically or conventionally produced (4). The problem of illegal addition of cheaper or low-quality meat from other animal species to meat products received large attention in 2013 as a result of the horse meat scandal (1, 5, 6). The general interest in detailed information on food has since grown and in particular the interest in authenticity and traceability of fresh meat and processed meat products has greatly increased. Accurate labeling is important to consumers for many reasons, such as the fact that some population groups do not consume specific types of animals for health, religious, and ethical reasons. Consumers are only enabled to make an informed purchasing decision if correct information about the nature of the product is given (3, 7).

Therefore, robust and reliable analytical methods must be available to detect and trace food fraud. For the identification of animal species, polymerase chain reaction based techniques and antibody-based methods have become established procedures (8, 9). The metabolomics approach is described as a new potential method for determining the authenticity of meat and meat products. In food analysis, metabolomics approaches aim at animal or plant based metabolites of hydrophilic and/or hydrophobic nature with a low molecular weight ≤ 1,000 Da (10, 11). By using proton nuclear magnetic resonance spectroscopy (^1^H NMR) it is possible to obtain a characteristic pattern of metabolites, referred to as “chemical fingerprint,” within a single NMR spectrum (12). The application of ^1^H NMR based metabolomics in the food sector has increased continuously in recent years, especially in combination with multivariate statistical methods such as principal component analysis (PCA) and linear discriminate analysis (LDA) (12–16). As a non—targeted approach, the entire spectral information of a sample allows for statements about the authenticity, but also about the origin of the sample. Thus, this approach is not focused on single compounds, but all information about a sample is collected (17, 18). There are a few ^1^H NMR studies related to meat, such as distinguishing beef from four countries by analyzing aqueous meat extracts (19). Another study focuses on the authentication of beef and horse meat using a low—field NMR spectrometer at 60 MHz to reveal differences in triacylglyceride signatures (20).

In a previously published paper we were able to demonstrate that analysis of the non-polar metabolites of meat by ^1^H NMR in combination with multivariate statistics allows for a differentiation of the meat species (21). Here, we combine a non-targeted ^1^H NMR based analysis of polar meat metabolites with a multivariate statistical approach to differentiate between various meat species. In order to obtain a classification model that contains information about both polar and non-polar metabolites, a mid-level data fusion was also performed.

## Materials and methods

### Samples and chemicals

A total of 419 raw meat samples were collected between the years 2018 and 2021. Most of the samples came from the state of Baden—Württemberg, Germany, and were taken by official food inspectors of the German Federal State of Baden Württemberg; also, some samples were bought at local supermarkets and butchers. The samples included 185 pork, 115 beef, 71 lamb, and 48 poultry (chicken and turkey) samples and involved a variety of cuts and mince. A total of 379 meat samples (175 pork, 105 beef, 61 lamb, and 38 poultry) were used for multivariate data analysis and establishment of the model. The remaining 40 samples (consisting of 10 pork, 10 beef, 10 lamb, and 10 poultry samples) were used for external validation (chapter 2.5.4). One chicken sample was purchased from a local butcher and was used as a control sample for the method validation. In addition to raw meat samples, 76 processed meat products of the meat species beef (19), pork (22), and poultry (23) were also analyzed (Supplementary Table 1). In addition, five processed meat products containing poultry/pork (4) and beef/pork (3) were used to test the robustness of the classification model against mixtures (Supplementary Table 3).

Sodium dihydrogen phosphate (≥99.0%), 3-(trimethylsilyl)-propionic acid-*d*_4_ sodium salt (TSP, 98.0% atom % D), and D_2_O (99.9% atom % D) were purchased from Merck (Darmstadt, Germany).

### Sample preparation

Bones, rind, subcutaneous fat, and innards were removed from the meat samples. Following mixing, the samples were freeze-dried and ground in a cryomill (SamplePrep6870 Freezer Mill, C3 Process and Analysis Technology GmbH, Haar, Germany). The ground, dry samples were stored in a freezer (−20°C) until use. The meat powder (500 mg) was extracted with 6 mL of water. After the samples were mixed on a test tube shaker (Multi Reax, Heidolph, Schwabach, Germany) for 10 min, samples were centrifuged at 3,000 rpm [relative centrifugal force (RCF), 1,690 x g] for 15 min. The aqueous supernatant was passed through a syringe filter (Chromafil Xtra PET −45/25, Macherey-Nagel, Düren, Germany) into a centrifuge tube. The 3 kDa ultrafiltration filters (Vivaspin^®^, Sartorius, Goettingen, Germany) were rinsed three times with 2 mL of water each to remove glycerol [10 min at 3,000 rpm, relative centrifugal force (RCF), 1,690 × g]. After the cleaning step, 800 μL of the meat extract was transferred to the 3 kDa ultrafiltration unit, and samples were centrifuged at 3,000 rpm [relative centrifugal force (RCF), 1690 × g] for 1.5 h. An aliquot of the obtained filtrate (500 μL) was mixed with 250 μL of 3 M sodium dihydrogen phosphate buffer (pH 6) and 75 μL of TSP (dissolved in D_2_O). A 600 μL-aliquot of this mixture was transferred to a 5-mm Boro 300-5-8 (Deutero, Bad Kreuznach, Germany) NMR tube.

### Method validation

For the method validation a control sample (chicken) was established in order to verify the sample preparation step including extraction, and measurement performance. First, the control sample was extracted five times on three consecutive days, and second, the extraction was carried out five times by another person. All extracts were analyzed as described below. In addition, the control sample was analyzed in each analytical series including aqueous extraction and ^1^H NMR measurement to ensure an issue free sample preparation.

### NMR spectroscopy

All spectra were recorded using the same parameters and under the same conditions. ^1^H NMR spectra were measured on a Bruker 400 MHz AVANCE III HD NanoBay spectrometer (Bruker Biospin GmbH, Reinstetten, Germany) equipped with a 5-mm BBI (broadband inverse) probe at 300 K. A Bruker automatic sample changer Sample Xpress (Bruker Biospin GmbH, Rheinstetten, Germany) was used. After a 5-min temperature adjustment, automatic tuning and matching and automatic shimming were applied. For each sample an automatic pulse calibration was performed. All ^1^H NMR spectra were recorded using the standard Bruker pulse program *noesygppr1d_d7.eba* with a relaxation delay (D1) of 4 s and an acquisition time of 8 s. ^1^H NMR parameters were as follows: 128 k time domain data points, 128 scans, 4 dummy scans, spectral width of 20.5617 ppm, size of FID (TD) 135168, digmod was baseopt, and receiver gain was 64. Processing was performed using the Bruker Biospin Topspin software (version 3.2): exponential window function was applied, a zero filling was performed (SI = 2TD) and line broadening was set to 0.3 Hz, followed by a Fourier Transformation, spectral phasing, and baseline correction. All spectra were referenced to the TSP signal at 0 ppm. To ensure spectrum quality, the full width at half maximum of the TSP signal was determined. A limit of 1.2 Hz was set and if this was exceeded, the analysis had to be repeated.

### Data analysis

#### Data reduction and pretreatment of the ^1^H NMR spectra

To reduce data and to provide input variables for statistical analysis, bucketing was performed. The spectral region in the range of 0.50–9.50 ppm was divided into 1,000 equal segments, followed by exclusion of the region of residual water (4.84–5.10 ppm). Spectra were normalized to the signal of TSP (−0.5 to 0.5 ppm). A pseudo-scaling effect was achieved by applying a log transformation. NMR data were analyzed using MATLAB version 2013b (The Math Works, Natick, MA, USA).

#### Multivariate statistical data analysis

The potential to predict the animal species of meat by analyzing ^1^H NMR data of polar meat metabolites was validated using a combination of established multivariate statistical tools: PCA with LDA, and multivariate analysis of variance within a cross-validation (CV) embedded in a Monte Carlo (MC) resampling approach. The following classification rule was set: a test set object was assigned to the class with minimum distance between test set object and respective class mean, that is, assignment according to the nearest class mean (NCM).

#### Model building PCA/LDA and MC embedded CV

A total of 379 meat samples was used to build and validate the prediction model; the model was built by using 90% of these samples, and 10% of the samples were used as internal test set. A PCA was performed in order to reduce the dimensions. PCA was followed by LDA to get a maximum of class separation. The quality was assigned by using NCM classification. The distance between the object of the test set and the class means of the model set was compared, and the group membership was assigned. To validate the predictivity of the PCA/LDA, a CV with ten randomly selected disjunct subsequent test sets was performed. To avoid any segmentation bias, CV was repeated 10 times with an MC resampling approach (MC = 10) always with a new random segmentation for each CV step. Lastly, the rate of correct and false class predictions was calculated for each class to set up a confusion matrix. With the help of the confusion matrix the measurement correctness of the CV is represented. In this matrix, information about the dependence of the true class and the class assigned by means of classification model is obtained.

#### External model validation

For an external validation approach, samples of each meat species that were not used to build the model were utilized. A test set of 40 samples (consisting of 10 pork, 10 beef, 10 lamb, and 10 poultry samples) were applied to the classification model, which was built from the 379 samples. Results of the assignment are indicated as distances of the samples to the model mean value (with a confidence interval of 95%) and as *p*-values supporting the decision whether a sample is beef, pork, lamb, or poultry.

#### Multiple univariate testing for spectral differences between meat species

To detect spectral regions that significantly differ between the different meat species the Kruskal-Wallis test as a non—parametric version of a one-way analysis of variance (ANOVA) was applied with a significance level of α = 0.001, which was Šidák corrected (24). It is evaluated whether the expectation values of the means of different statistical samples are different. Different from ANOVA, the Kruskal-Wallis test is not based on a Gaussian distribution of the data. Because the Kruskal-Wallis test operates on a single variable, it has to be applied multiple times, scanning intensities at each individual ppm-value for spectral analysis (15, 25).

#### Identification of possible marker compounds for the discrimination of meat species *via* polar metabolites

The PCA/LDA score plot and loading plot were plotted using the MATLAB version 2013b (The Math Works, Natick, MA, USA). By analysis of the loading plots, variables were extracted that affect the discrimination or separation in the score plot most strongly. For clarity, four two-class models (pork/beef, pork/lamb, lamb/beef, and poultry/non-poultry) were used.

#### Mid-level data fusion

Fusion of the ^1^H NMR data of the analyses of the non-polar and polar metabolites was investigated with a mid-level approach using MATLAB version 2013b with Statistical Toolbox (The Math Works, Natick, MA, USA). First, the data sets consisting of 379 samples were separately subjected to data pretreatment (bucketing, solvent exclusion, normalization, and log transformation). PCA was then used to perform data reduction and simultaneous selection of relevant variables from each data matrix, forming the respective scores. The scores obtained by PCA were fused in the next step, resulting in a joint dataset. This dataset was used to construct the LDA.

## Results and discussion

### Sample preparation and ^1^H NMR signal assignments

**Figure 1:**
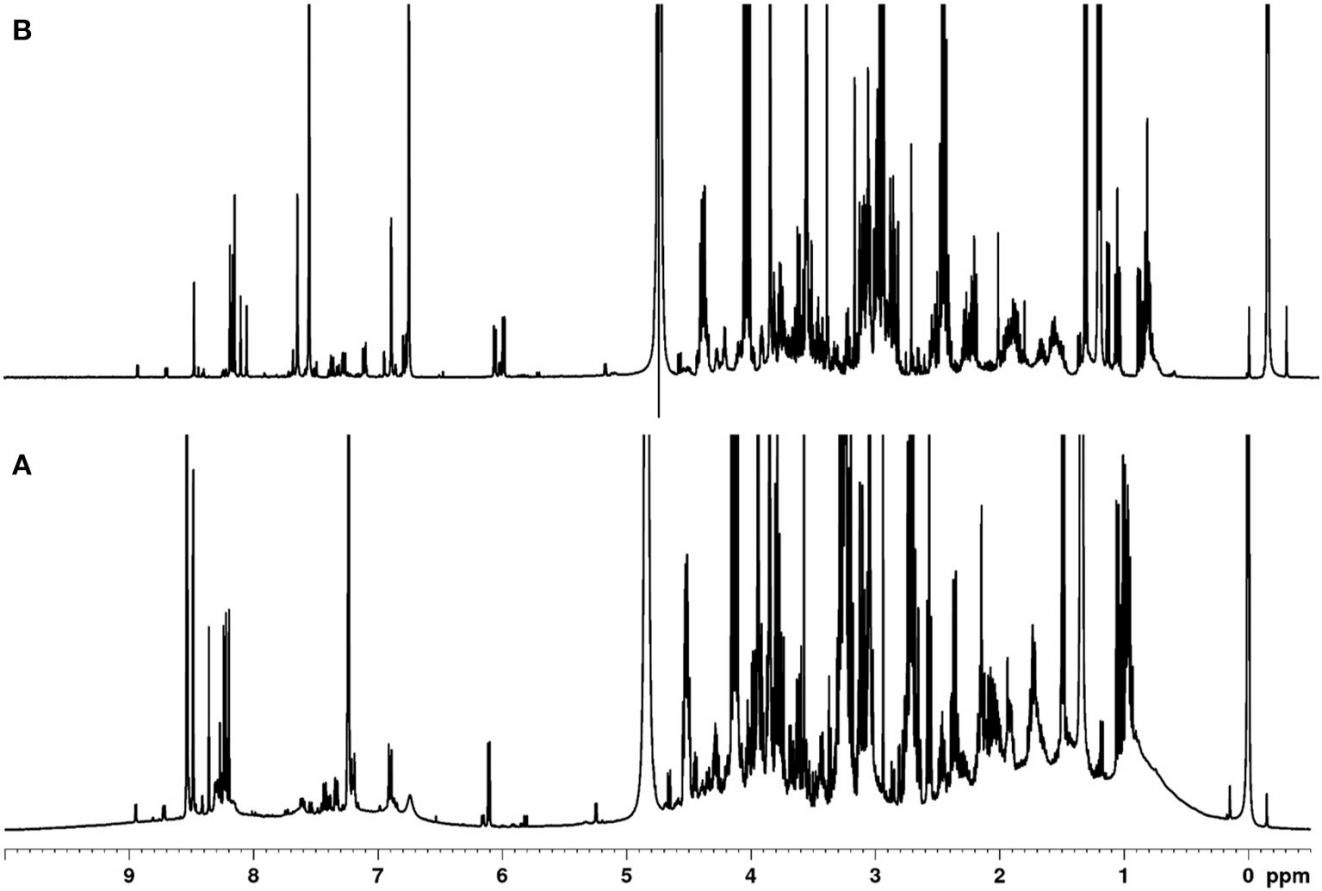
Comparison of the 400 MHz-^1^H NMR spectra of a polar meat extract without **(A)** and after protein removal **(B)**.

Polar metabolites in meat derive from numerous low molecular weight compound classes with high chemical diversity. These classes include amino acids and their derivatives, organic acids, carbohydrates, purine derivatives, imidazole dipeptides, and quaternary ammonium compounds (QAC) (26). Various extraction protocols are described in the literature to capture polar meat compounds, but these often only focus on a single class of compounds, such as amino acids or imidazole dipeptides (27–30). Here, an extraction procedure was developed that allows for simultaneous extraction and detection of the majority of polar metabolites. Although the majority of low molecular weight metabolites should be included, polymers may interfere with the analysis. Thus, the initial focus was on the removal of proteins, since these reduce spectra quality. Figure 1 shows the ^1^H NMR spectrum of the polar metabolites of meat with and without protein removal. Without protein removal (Figure 1A), broad signals occur in the range of 0.5–5.0 and 6.5–9.0 ppm, which can be assigned to co-extracted, non—precipitated proteins. Because these signals largely vary depending on the individual sample, a reasonable integration of the low molecular weight metabolite signals was not possible as also described in literature (31, 32).

**Figure 2:**
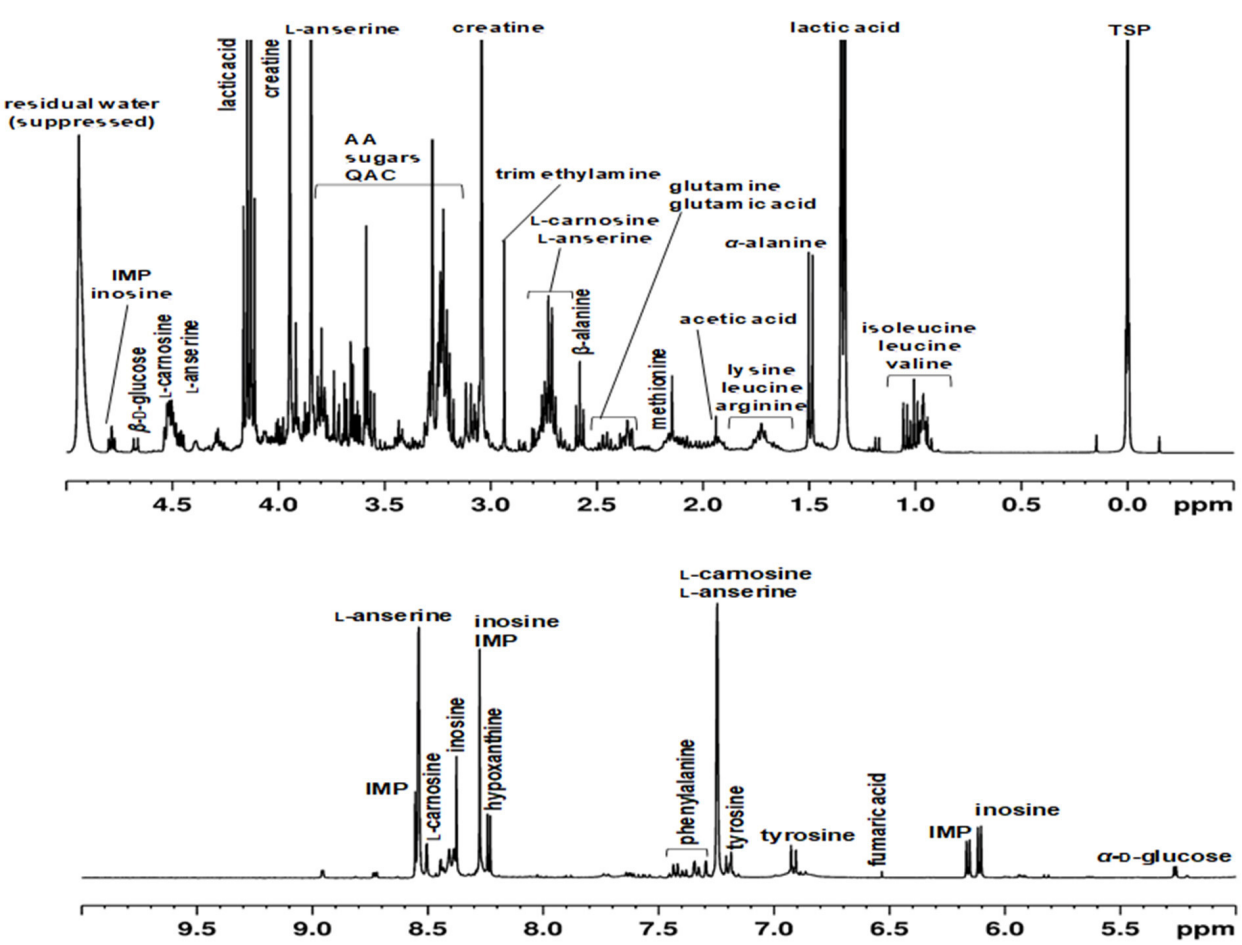
Representative ^1^H NMR spectra of an aqueous extract of a meat sample (poultry) recorded in H_2_O/D_2_O (10:1, v/v) using a 400 MHz spectrometer. Chemical shifts were calibrated against the TSP signal at 0 ppm. The residual water signal (4.84–5.10 ppm) was suppressed using the *noesygppr1d_d7.eba* experiment. IMP, inosine monophosphate; AA, amino acids; QAC, quaternary ammonium compounds.

In the past, approaches have been described, in which protein precipitation occurs before or after the actual extraction (31–33). Also, depending on the extraction solvents, they may already be suitable to precipitate proteins (23, 34, 35). Here, different methods of protein removal in polar meat extracts were investigated and evaluated in terms of reproducibility, degree of protein removal, associated baseline smoothing, and sharpness of signals. Ultrafiltration using a 3 kDa filter at room temperature was found to be the most effective, gentle and reproducible method for protein removal. The ultra-filtrate, which was obtained within 1.5 h, was directly measured by an optimized ^1^H NMR spectroscopic procedure (Figure 1B). Assignment of ^1^H NMR signals was performed according to existing literature, interpretation of two-dimensional NMR spectroscopy (*hsqcetgp, cosygpppqf, mlevphpr.2*) data, and spectra of commercially available standard compounds. The spectral region between 0.0 and 5.0 ppm shows signals of a large number of metabolites (Figure 2A). Numerous signals with multiplet structures were detected, complicating their identification. Prominent signals represent lactic acid, α-alanine, creatine, L-anserine, and L-carnosine. Especially in the 3.0–3.8 ppm range, many different compounds were detected, such as the α*-*CH group of α-amino acids, multiplet signals of imidazole dipeptides, and sugar signals. Also, signals of QAC such as carnitine, *O*-acetyl-L-carnitine, betaine, and choline are located in this region.

Figure 2B shows the spectral region of 5.0–10.0 ppm, in which predominantly signals of aromatic metabolites such as phenylalanine and tyrosine are found. Also, proton signals of the imidazole ring of L-anserine und L-carnosine are present in the low field. Signals of inosine, inosine monophosphate (IMP), and hypoxanthine are also located in the aromatic region of the ^1^H NMR spectrum. All three metabolites are involved in postmortem energy metabolism of the muscle and are critical for the flavor development of meat. Adenosine triphosphate (ATP) formed by anaerobic glycolysis is irreversibly degraded during the process of slaughtering *via* adenosine diphosphate and adenosine monophosphate to IMP and ammonia. IMP is further degraded by dephosphorylation to inosine, which is subsequently hydrolyzed to hypoxanthine and ribose (22, 36).

### Univariate data analysis

**Figure 3:**
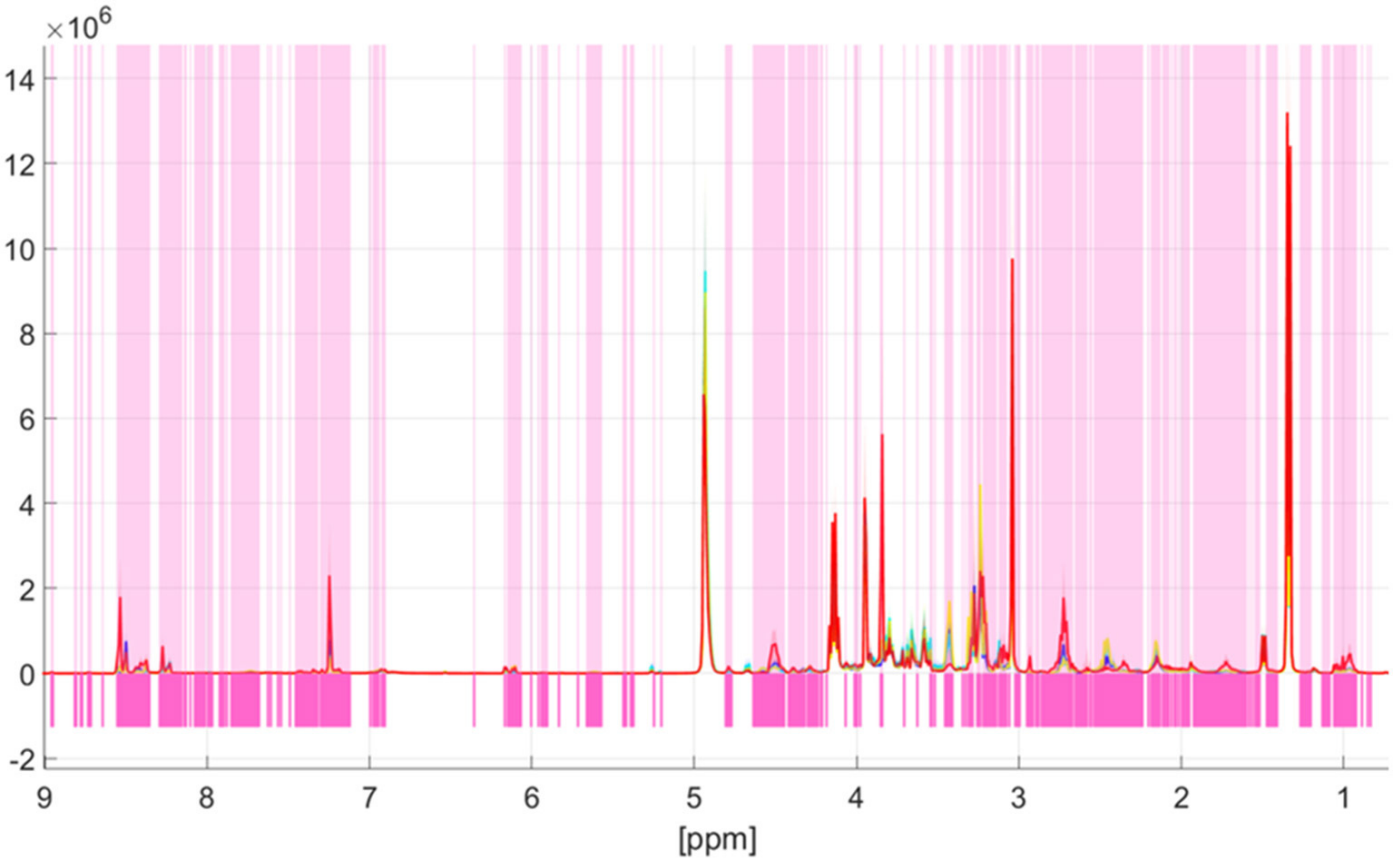
Identification of spectroscopic regions that are responsible for the discrimination of the meat species pork, lamb, beef, and poultry. The *p*-values according to the Kruskal-Wallis test are color coded (white: high *p*-value, magenta: low *p*-value). Low *p*-values indicate spectral regions containing information that contribute to the discrimination. Meat species are marked as follows: poultry—red; beef—turquoise; lamb—yellow; pork—blue. The thick line describes the respective mean value of each individual group, the colored areas around the respective mean value show ranges of variation within a group.

Univariate data analysis based on the Kruskal-Wallis test showed spectral differences between the four meat species pork, beef, lamb, and poultry. Significant differences of each bucket between species groups were analyzed with a confidence level set to 99.9%. A Šidák correction was used to avoid the problem of multiple testing. The previously selected significance level α = 0.001 was corrected by the formula 1-(1-α)^1/k^, with k being the number of buckets after pretreatment (k = 967) (24). The corrected significance level was α_SID_ = 1.03^*^10^−6^. A bucket was considered significantly different when the *p*-value was below α_SID_. Figure 3 shows the results of the Kruskal-Wallis test, with pink bars indicating buckets, which are different between the four groups pork, beef, lamb, and poultry. White areas indicate high *p*-values which demonstrate spectral regions that do not contain information on the animal species (15). Obviously, many areas feature differences, in both high field and low field spectral regions. Thus, there is a large number of metabolites that differ among the meat species. Signal regions that do not reveal differences include, for example, the metabolites lactate (1.34 ppm; 4.12 ppm), α-alanine (1.48 ppm), and creatine (3.04 ppm, 3.97 ppm). Similarly, the signals close to the residual water signal, which represent the anomeric protons of α-glucose at 5.24 ppm and β—glucose at 4.64 ppm, respectively, are not highlighted in pink. In general, the differences found in the ^1^H NMR spectra were typically attributed to subtle intensity differences and not due to the presence or absence of class specific signals. Which metabolites were actually responsible for the separation in the classification model will be shown later in the loading plot (37).

### Classification of meat species by ^1^H NMR spectroscopy and combined multivariate statistical analysis

For the multivariate statistical analysis all ^1^H NMR spectra had to be automatically phased and baseline corrected. The range between 0.50 and 9.50 ppm was chosen for bucketing and was divided into 1,000 equal buckets. Following bucketing, the residual water signal (4.84–5.10 ppm) was excluded, and data was normalized in order to compensate potential differences during sample preparation. Normalization was performed to the internal reference standard TSP (−0.5 to 0.5 ppm), because formation of complexes with proteins was judged to be unlikely due to previous protein removal (38). In addition, univariate data analysis showed that the TSP signal was not different in the 379 samples. As the last data pretreatment step, log transformation was performed to achieve a pseudo-scaling effect and reduce differences between large and small values in the data. The transformation reduces larger values in the data set more than small values (39, 40). With the now pretreated data, the classification model was subsequently formed and validated.

Because the number of variables (bucket data) was extremely high for subsequent statistical treatments, PCA was used for dimension reduction. LDA was applied to the PCA scores in order to identify the multivariate subspace for maximum class (meat species) separation (41, 42). For LDA, the scores of the first 16 dimensions of the PCA were used, describing 98.9% of the variance of the data. The first three principal components already represent 97.7% of the total variance of the data. In order to judge the predictive power and reliability of the model, the statistical model was validated: internal validation was carried out by using the 10 fold-CV (ten randomly selected subsequent test sets) approach, for which the data were divided into a training set (used to build a model) and a test set (used to test the prediction ability). With completion of CV, each ^1^H NMR spectrum was in the test set once. To overcome the potential risk of segmentation bias, the CV was carried out multiple times using a MC resampling approach (14, 15). At the beginning, shuffling of the complete data set was performed followed by a repeated 10 fold-CV. This step results in a new random segmentation into test and training samples. In the present work ten MC runs were performed, resulting in 100 models with associated confusion matrices. These 100 matrices were combined into a single confusion matrix. This confusion matrix was the final result of the embedded MCCV, which determines the predictive accuracy of the model. Assignment of test set samples to meat species classes was carried out by comparing distances between test objects and class means (NCM method) (12).

**Figure 4:**
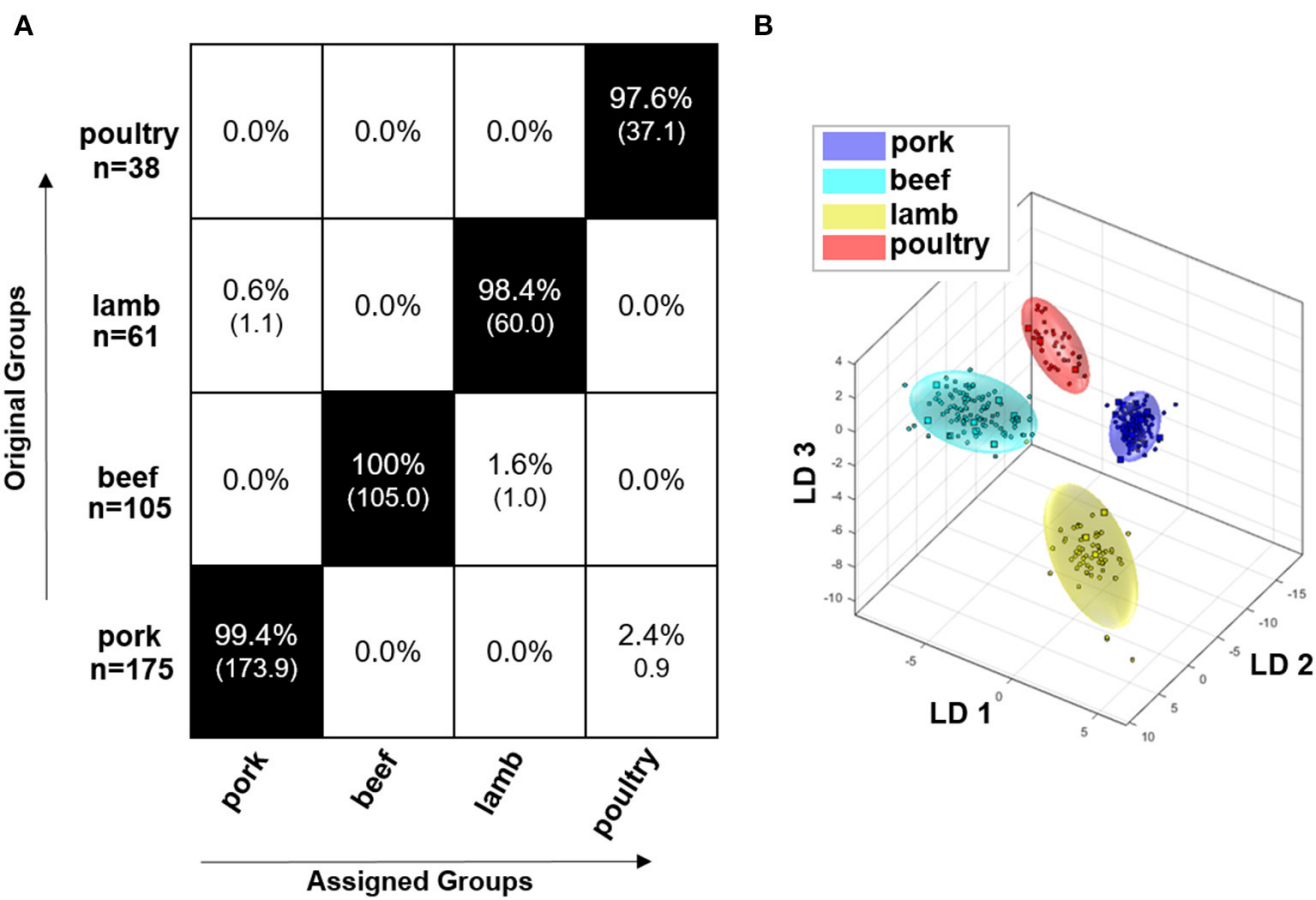
Results of the Monte Carlo embedded cross-validation on the performance of the obtained classification model of PCA/LDA to predict the species of meat using ^1^H NMR data of polar meat metabolites. **(B)** shows the discrimination space of a cross validation step. The training set for model building of each class is symbolized by its 95% confidence ellipsoid, and the test set samples are indicated as circles. **(A)** shows the confusion matrix of the embedded MCCV. The x-axis and the y-axis show the assigned class and the correct class, respectively. The confusion matrix shows the classification assignment in numbers and the accuracies about the probability of the prediction result in percent. Experiment used: *noesygppr1d_d7.eba*; 0.50–9.50 ppm; 1,000 buckets; exclusion of the residual water signal (4.84–5.10 ppm); normalization: −0.50 to 0.50 ppm; log transformation; PCA dimensions: 14; 10 CV; 10 Monte Carlo runs.

Figure 4A shows the result of the embedded MCCV as a confusion matrix of the obtained classification model for the polar metabolites of the raw meat samples. It can be seen that the model that is based on polar metabolites is suitable for the differentiation of the meat species pork, beef, lamb, and poultry. The confusion matrix demonstrates that the accuracy of the assignment of the respective meat species to the correct class is between 97.6 and 100.0%. Accordingly, all meat species were assigned with a high accuracy. Figure 4B illustrates the discrimination space of one CV step. The training set for model building of each class is symbolized by its 95% confidence ellipsoid, the test set samples are marked as circles. The groups form clusters with no overlapping areas. It is thus possible to represent the four meat species in a multi-class model and it is not necessary to resort to a binary model due to confounding.

### Classification-relevant spectral regions and potential chemical markers

**Figure 5:**
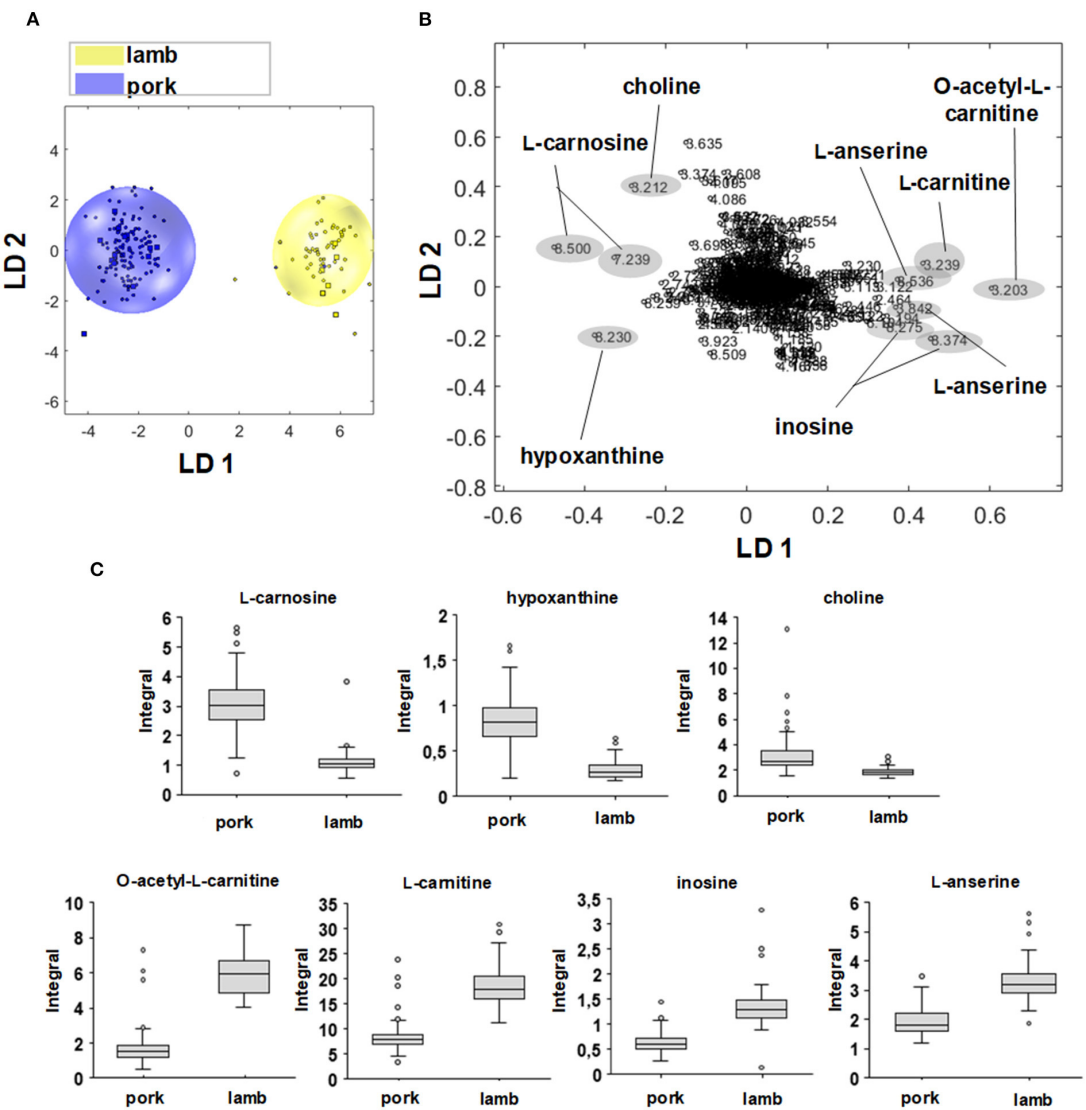
**(A)** Two-dimensional PCA/LDA score plot of the sample groups lamb (yellow) and pork (blue). **(B)** Associated loading plot with the 967 buckets used. Buckets with the highest positive or highest negative values along LD 1 are marked and correspond to signal regions that are more distinct in the respective sample group. **(C)** Box-Whisker-Plots of metabolites that are relevant for the discrimination of pork vs. lamb. Buckets that showed an effect on discrimination in the loading plot were used for the Box-Whisker-Plot. Box-Whisker-Plots of L-carnosine, hypoxanthine, and choline demonstrate higher concentrations in beef meat. Box Whisker-Plots of L-anserine, inosine, L-carnitine, and *O*-acetyl-L-carnitine demonstrate higher concentration sin lamb meat.

Regions that are relevant for classification can be extracted from PCA/LDA loading plots, which indicate buckets that mostly affect clustering of the respective sample (37, 43). In contrast, the Kruskal-Wallis test (Section 3.2) only indicated spectral differences between meat species but not, which metabolites (or spectral regions) are actually responsible for the discrimination into clusters. For clarity, the loadings are presented in two-class models (Figure 5). In order to illustrate the analytically obtained results, the relative distribution in the sample set was plotted using the Box-Whisker-Plot to show the location and dispersion of the values for some classification—relevant metabolites. In the following, a total of four different two-class models (pork/beef, pork/lamb, lamb/beef, and poultry/non-poultry) was used.

Figure 5A shows the two-dimensional PCA/LDA score plot for the differentiation of pork and lamb meat samples *via* the polar metabolites, Figure 5B the corresponding loading plot. The lamb and pork samples are visualized as yellow and blue dots, both groups show separated clusters along the first linear discrimination function (LD). Variables that are far from the origin with respect to the plotted LD affect the model. Accordingly, variables that are close to the origin are not significant for the model with respect to the plotted LD (37). For the negative loading values along LD 1, which correlated with the scores of the pork samples, the buckets 8.500 and 7.239 ppm showed high negative loading values. Both were assigned to L-carnosine, which is present in significantly higher concentrations in pork than in lamb meat. This was confirmed by the Box—Whisker-Plot in Figure 5C. However, the variance of the values for the pork group was greater than for the lamb group. In addition, the bucket at 8.230 ppm was assigned to hypoxanthine, and choline was identified as being responsible for the bucket at 3.212 ppm. The Box-Whisker-Plots indicated increased concentrations of both metabolites in pork meat. Again, a larger variability of hypoxanthine levels in pork samples was observed. The positive loading values along LD 1 correlated with the lamb samples; the buckets 3.203 and 3.239 ppm correspond to signals of *O*—acetyl-L-carnitine and L-carnitine. The buckets at 8.275 and 8.374 ppm, respectively, were assigned to inosine, the buckets at 3.842 and 8.536 ppm to L-anserine. The Box—Whisker-Plots show an increased concentration in lamb meat for the four metabolites.

Buckets that were assigned to QAC (L-carnitine, choline, *O*—acetyl-L-carnitine), imidazole dipeptides (L-anserine, L-carnosine), and degradation products of ATP (inosine, hypoxanthine) were demonstrated to be involved in the classification of beef/lamb, beef/pork, and/or poultry/non-poultry (Supplementary Figure 1; Supplementary Table 2). In literature, especially levels of imidazole dipeptides and their ratios were described as being species-dependent (29, 44–46).

### Mid-level data fusion and method validation

**Figure 6:**
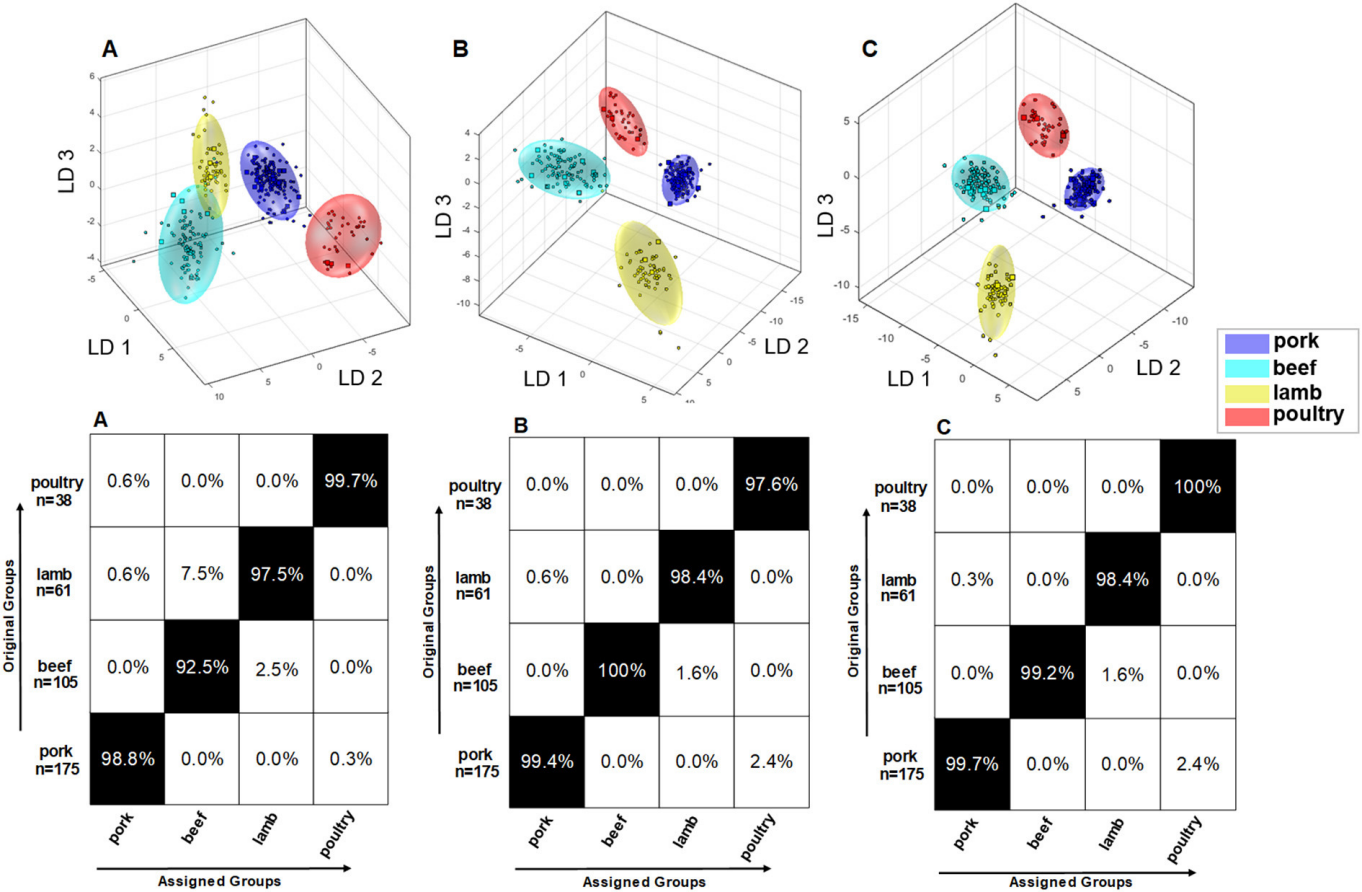
**(A)** Results of embedded MCCV on the obtained classification model of PCA/LDA to predict the animal species *via* the ^1^H NMR spectra of nonpolar metabolites according the method of (21). Used experiment *zg30*; 0.50–6.80 ppm; 2,000 buckets; exclusion of the residual water signal (4.84–5.10 ppm) and methanol signal (3.33–4.40 ppm); normalization: 1.50–4.050 ppm; log transformation; PCA dimensions: 16;10 CV; 10 Monte Carlo runs. **(B)** Results of embedded MCCV on the obtained classification model of PCA/LDA to predict the animal species i the ^1^H NMR spectra of the polar metabolites. Used experiment *noesygppr1d_d7.eba*; 0.50–9.50 ppm; 1,000 buckets; exclusion of the residual water signal (4.84–5.10 ppm); normalization: TSP (−0.5 to 0.5 ppm); log transformation; PCA dimensions: 16; 10 CV; 10 Monte Carlo runs. **(C)** Results of embedded MCCV on the obtained classification model of PCA/LDA formed by the previously performed mid-level data fusion. By applying mid-level data fusion, the respective scores generated by the previously performed PCA were combined from both data on polar and non-polar metabolites, and a new classification model was created.

In a previously published paper, an attempt was made to build a classification model based on non-polar metabolites of meat (21). By combining data on polar and non-polar metabolites, a mid-level data fusion was used to establish a new classification model that contains maximum metabolite information. Figure 6 shows three different models with associated confusion matrices. Data model A describes the PCA/LDA from 379 samples that were analyzed for their non-polar metabolites. Accordingly, data model B is constructed from the same 379 samples that, however, where analyzed for their polar metabolites (Figure 4). By applying mid—level data fusion, the scores from previously performed PCAs (both extraction methods) were combined and used to build a new classification model (47, 48). Data model C describes the PCA/LDA that was applied to the fused PCA scores. The confusion matrices in Figure 6C show that the classification results can be improved after data fusion. However, because the classification accuracy already ranged between 92.5 and 100.0% in data models A and B, data fusion only resulted in moderate improvements. The beef samples were correctly assigned with a classification accuracy of 92.5% using data model A. The remaining 7.5% of the samples were assigned as lamb. Also, in model A it is evident that the beef cloud (turquoise) and lamb cloud (yellow) slightly touch each other. In contrast, by using the model based on polar metabolites (model B) all beef samples were correctly assigned. In model B, the clusters of beef and lamb are also much better separated than in model A. Data fusion allowed for a correct assignment of 99.2% of all beef samples (model C). Classification accuracy improved for lamb based meat by combining data on polar and non-polar metabolites. In contrast, assignment of poultry based meat was slightly better when the non-polar compounds were analyzed (model A) as compared to the analysis of polar metabolites (model B). Mid-level data fusion (model C) allowed the poultry samples to be assigned with a classification accuracy of 100%. Also, due to the lower dispersion of the samples within an animal species the limits of the 95% confidence interval were automatically reduced, and the clusters became smaller. This can nicely be seen for the poultry samples (red) in model C as compared to these samples in model A, but also for the beef samples (turquoise) and the pork samples (blue). As a result, by combining the data there is an improved spatial separation of the clusters, and the absolute distances between the clusters increase. As previously mentioned, application of new samples to the built classification model was tested, too. Ten new samples of each species were analyzed as an external validation set for correct assignment. For each of these ten test samples ^1^H NMR spectra of the lipophilic and hydrophilic extracts were recorded. For all four species, all ten test samples were correctly assigned. Supplementary Figure 2 shows the cluster models including the ten test samples, which are highlighted as red stars. All samples met the specified significance level *p*-value ≥ 0.05. Thus, combining the different sources of information is reflected in an improvement of the multivariate system.

### Classification of processed meat products

**Figure 7:**
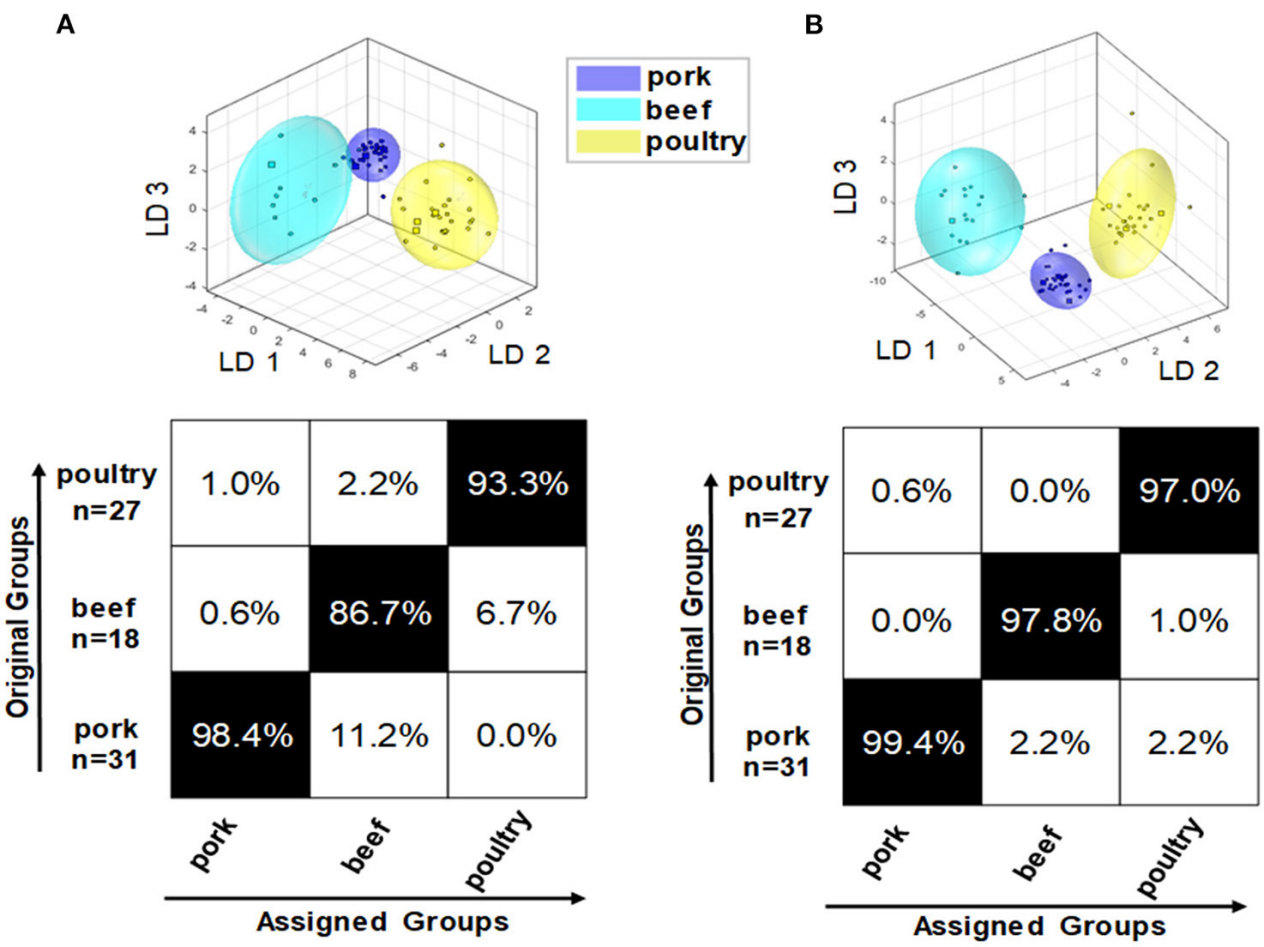
Results of embedded MCCV on the obtained classification model of PCA/LDA to predict the species of processed meat products *via* the ^1^H NMR spectra of the polar metabolites **(A)** as well as after mid-level data fusion **(B)**.

In addition to raw meat, processed products based on beef, pork, and poultry meat were studied. All 76 processed meat products (pork: 31; beef: 18; poultry: 27) were analyzed for their polar and non-polar metabolites followed by PCA/LDA. To potentially increase classification and prediction accuracy, a mid-level data fusion was also performed using the PCA scores of the two extraction procedures. Data pretreatment was performed as for the fresh meat samples. Figure 7 shows the confusion matrix of the polar metabolite classification model (A). For the PCA/LDA, the scores of the first 14 dimensions of the PCA were used, describing a total of 93.4% of the variance in the data. The confusion matrix shows that the model that is built on polar metabolites is generally suitable for differentiating the species of pork, beef, and poultry in processed meat products. However, the confusion matrix also shows that the accuracy of the assignment varies between animal species. For beef based samples, the classification accuracy was as low as 86.7% with some samples being falsely classified as pork. Also the cluster model shows that the clusters of pork (blue) and cattle (turquoise) overlap slightly. Aside from that, a strong dispersion of the beef product data can be observed, clearly increasing the limits of the 95% confidence interval. This may be due to the limited number of beef based samples, which cover only a small variety of this product class. In contrast, the accuracy for the pork based products was high, 98.4%, and acceptable for the poultry-based products, 93.3%, too. The model of the non-polar metabolites can be seen in previously published study (21). For the PCA/LDA, the scores of the first 14 dimensions of the PCA, which describe 99.4% of the variance in the data, were used. Again, the differentiation of the three meat species was generally possible. The confusion matrix showed that the accuracy of assignment is ~97.0% for all three groups, thus being more accurate than the classification model that is based on polar metabolites. Also, the different clusters were completely separated from each other. However, particularly in the cluster representing beef based products the individual data points were highly scattered, increasing the 95% confidence interval. Combining information of both approaches (polar and non-polar extraction) in a mid-level data fusion results in data that are shown in Figure 7B. Overall, the fusion did not improve classification accuracies, which are similar to the results of the model that is based on non-polar metabolites. A minor improvement was seen in the scatter of the individual data points of the beef based samples, thus reducing the limits of the 95% confidence interval. Therefore, it can be stated that the primary goal of increasing the prediction accuracy of the classification was not achieved by mid-level data fusion. However, the accuracies of the individual models were already in a comparably high range.

**Figure 8:**
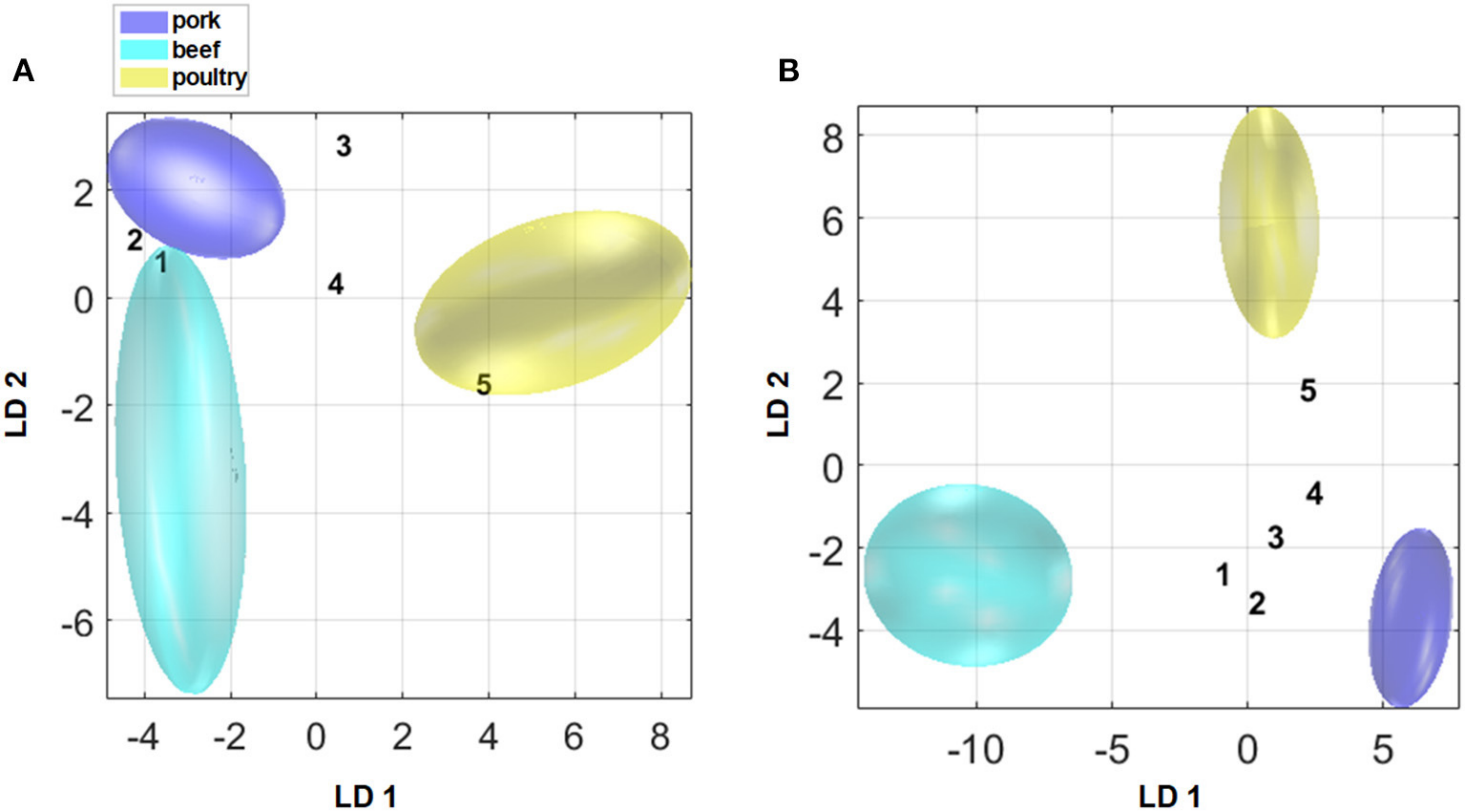
Illustration of the addition of five processed meat products that contain poultry and pork meat (samples 3–5) or beef and pork meat (samples 1 and 2) as new samples to the two models **(A)** based on polar metabolites, **(B)** classification model after mid-level data fusion).

In order to test the robustness and reliability of both the model based on polar metabolites and the combined model, five processed meat products consisting poultry/pork and beef/pork (Supplementary Table 3) were added to both models as a new sample set. Figure 8 shows that sample number 5 was assigned within the 95% confidence interval to the poultry group in the model that is based on polar metabolites (A) although the sample contains 9% of pork meat. In the previously published study on non-polar metabolites, the robustness of the non-polar model was also tested with the same five samples. Also, sample number 5 was assigned within the 95% confidence interval to the poultry group despite containing 9% of pork meat. The samples 1, 2, 3, and 4 were not assigned to any of the groups in the model that is based on non-polar metabolites. However, in these samples the composition of the two meat sources was more balanced than in sample 5 (21). By using the model that is based on polar metabolites, samples 1 and 2 were assigned within the 95% confidence interval of the beef group (Figure 8A). By applying the model that is based on data fusion, all samples were—correctly—not assigned to any of the groups (Figure 8B). These results were confirmed by the corresponding *p*-values, being smaller than 0.05 (Supplementary Table 3). Thus, data fusion results in a model that is more robust against mixtures of meat species in products. However, in the future more samples have to be added to the model. By increasing the number of samples, the robustness of the model will also be achieved.

## Data availability statement

The raw data supporting the conclusions of this article will be made available by the authors, without undue reservation.

## Author contributions

CD: conceptualization, methodology, investigation, laboratory work, and writing and editing. RK: conceptualization, editing, and supervision. TK: conceptualization, methodology, editing, and supervision. MB: conceptualization, methodology, writing and editing, and supervision. All authors contributed to the article and approved the submitted version.

## Funding

We acknowledge support by the KIT-Publication Fund of the Karlsruhe Institute of Technology.

## Conflict of interest

The authors declare that the research was conducted in the absence of any commercial or financial relationships that could be construed as a potential conflict of interest.

## Publisher's note

All claims expressed in this article are solely those of the authors and do not necessarily represent those of their affiliated organizations, or those of the publisher, the editors and the reviewers. Any product that may be evaluated in this article, or claim that may be made by its manufacturer, is not guaranteed or endorsed by the publisher.
