# Supplementary material for: Differentiation of meat species of raw and processed meat based on polar metabolites using 1H NMR spectroscopy combined with multivariate data analysis
Source: Front Nutr. 2022 Sep 30;9:985797. doi: 10.3389/fnut.2022.985797 (PMC9566576; doi:10.3389/fnut.2022.985797)
Supplement: Supplementary file 1 [file Data_Sheet_1.docx]

**Differentiation of meat species of raw and processed meat based on polar metabolites using ^1^H NMR spectroscopy combined with multivariate data analysis**

**Christina Decker^1,3^, Reiner Krapf^2^, Thomas Kuballa^3^, Mirko Bunzel^1^***

^1^Karlsruhe Institute of Technology (KIT), Department of Food Chemistry and Phytochemistry, Adenauerring 20A, D-76131 Karlsruhe, Germany

^2^ Bosch Power Tools, Max-Lang-Straße 40-46, D-70771 Leinfelden-Echterdingen, Germany

^2^ Chemisches und Veterinäruntersuchungsamt (CVUA) Karlsruhe, Weißenburger Straße 3, D‑76187 Karlsruhe, Germany

*** Correspondence:**Corresponding Author
mirko.bunzel@kit.edu


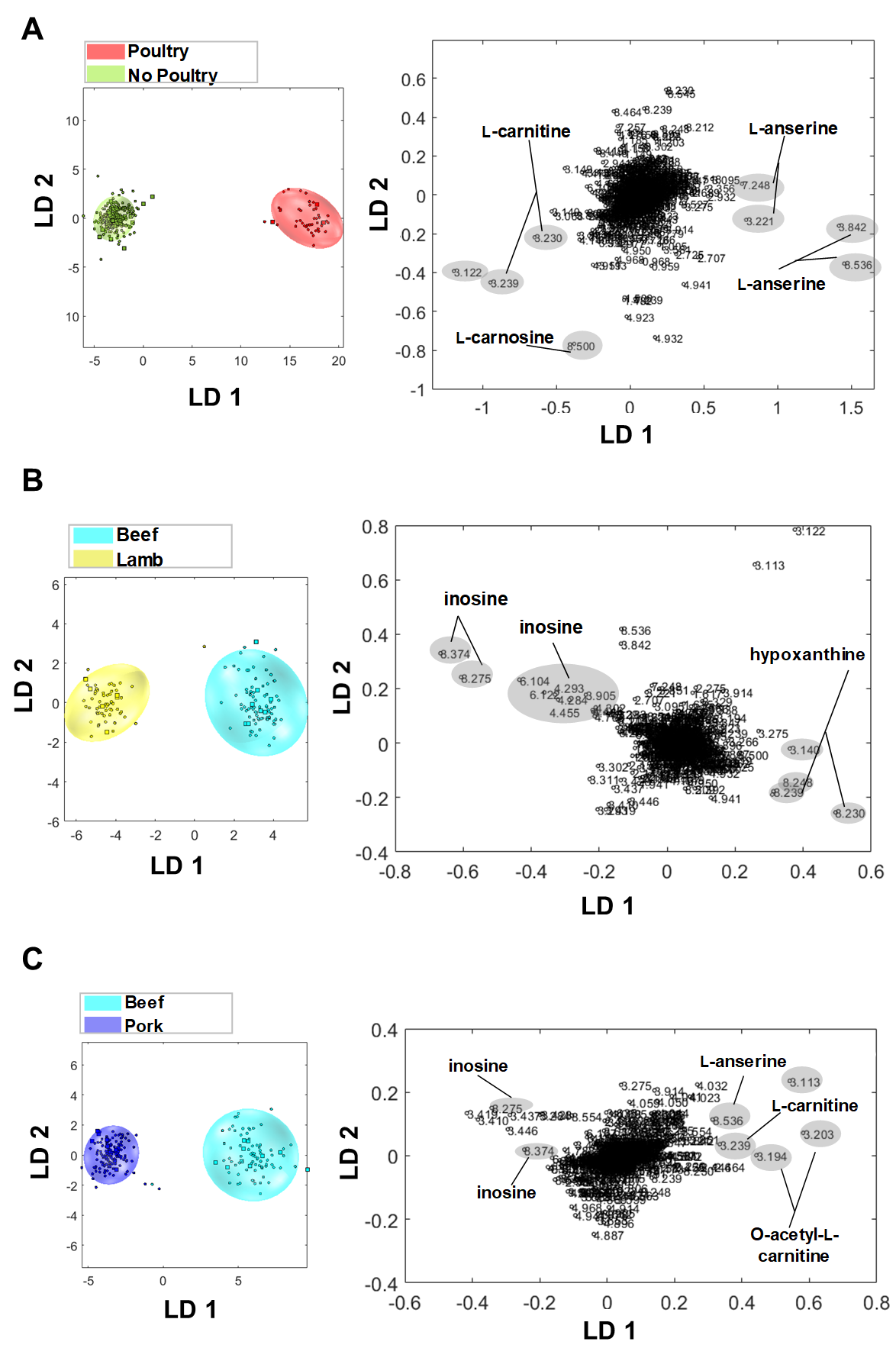


**Figure S1**: Two-dimensional PCA/LDA score plots of three different two-class models (**A:** poultry/non-poultry, **B:** beef/lamb, and **C:** beef/pork) and the associated loading plots with the 967 buckets used. The buckets with the highest positive or highest negative values along LD 1 (linear discrimination function) are marked grey and correspond to signal regions that are more distinct in the respective sample group.


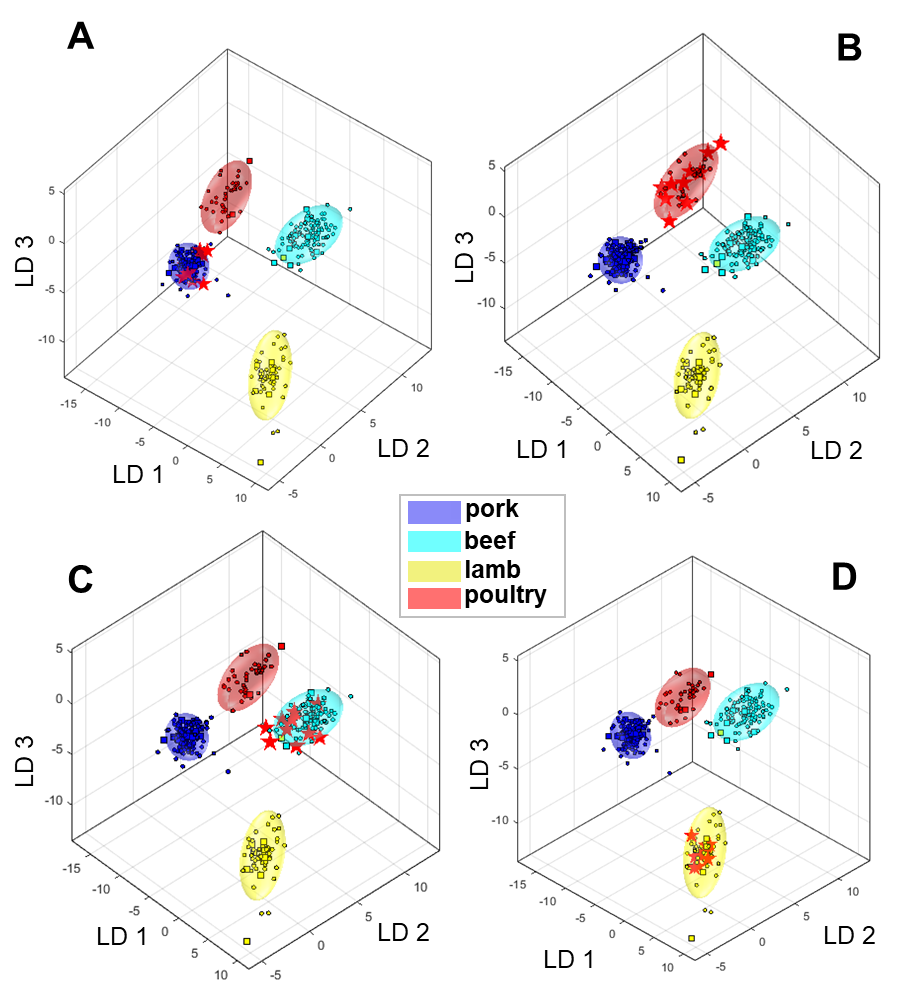


**Figure S2:** Results of the external validation of the classification model after mid-level data fusion. The discrimination spaces of a cross-validation step are shown. The training set for model building of each class is symbolized by its 95 % confidence ellipsoid, and the external validation set samples (n=10 per class) are indicated as red stars. **A:** external validation for the pork class, **B:** external validation for the poultry class, **C:** external validation for the beef class, **D:** external validation for the lamb class. LD: linear discriminate function

**Table S1:** Sample list of 76 processed meat sample, consist of one species each (poultry, beef, pork).

| number | name |
| --- | --- |
|  | **poultry** |
| 1 | salami light |
| 2 | bologna sausage |
| 3 | organic ham |
| 4 | chicken salami |
| 5 | ham |
| 6 | hunting sausage |
| 7 | bologna sausage |
| 8 | bologna sausage paprika |
| 9 | mortadella |
| 10 | paprika mortadella |
| 11 | poultry hunting sausage |
| 12 | turkey breast |
| 13 | poultry bratwurst |
| 14 | chicken salami |
| 15 | chicken breast fillet |
| 16 | turkey breast fillet |
| 17 | chicken breast fillet paprika |
| 18 | poultry wiener sausage |
| 19 | poultry mortadella |
| 20 | chicken breast fillet |
| 21 | chicken breast fillet |
| 22 | poultry bologna sausage |
| 23 | chicken breast |
| 24 | turkey breast grilled |
| 25 | chicken salami |
| 26 | mini sausage |
| 27 | chicken salami |
|  | **beef** |
| 28 | sucuk |
| 29 | roast beef fried |
| 30 | corned beef |
| 31 | bresaola black angus |
| 32 | corned beef |
| 33 | beef salami |
| 34 | irish angus beef burger |
| 35 | Beef salami |
| 36 | roast beef |
| 37 | smoked beef |
| 38 | pastrami brisket |
| 39 | beef sausage |
| 40 | beef mettwurst lean |
| 41 | Organic beef salami |
| 42 | beef cross rib |
| 43 | beef wiener |
| 44 | bresaola Punta d'Anca |
| 45 | roast beef with pepper |
|  | **pork** |
| 46 | bavarian weisswurst |
| 47 | jamon |
| 48 | salami |
| 49 | mortadella paprika |
| 50 | bologna sausage |
| 51 | bologna sausage pork |
| 52 | country ham |
| 53 | bavarian leberkäse |
| 54 | krakowska |
| 55 | thuringia sausage |
| 56 | organic ham |
| 57 | organic bacon |
| 58 | thuringia sausage |
| 59 | bratwurst |
| 60 | liver sausage |
| 61 | ham |
| 62 | bologna sausage |
| 63 | salami |
| 64 | landjäger |
| 65 | ham |
| 66 | beer ham |
| 67 | bockwurst |
| 68 | ham |
| 69 | beer ham |
| 70 | german sülze |
| 71 | salami milano |
| 72 | wiener |
| 73 | prosciutto cotto |
| 74 | mettwurst |
| 75 | beer ham |
| 76 | bologna sausage pork |

**Table S2:** Identified polar metabolites of meat and their impact on meat species discrimination

| Bucket ppm | Multiplicity | Metabolite | Significant for |
| --- | --- | --- | --- |
| 3.212 | s | choline | Lamb/Pork |
| 8.230 | s | hypoxanthine | Lamb/Pork |
| 8.500 | s | L-carnosine | Lamb/Pork |
| 7.239 | s | L-carnosine | Lamb/Pork |
| 8.275 | s | inosine | Lamb/Pork |
| 8.374 | s | inosine | Lamb/Pork |
| 3.239 | s | L-carnitine | Lamb/Pork |
| 3.203 | s | *O*-acetyl-L-carnitine | Lamb/Pork |
| 3.842 | s | L-anserine | Lamb/Pork |
| 8.536 | s | L-anserine | Lamb/Pork |
|  |  |  |  |
| 8.275 | s | inosine | Beef/Pork |
| 8.374 | s | inosine | Beef/Pork |
| 3.410 | - | unknown | Beef/Pork |
| 3.419 | - | unknown | Beef/Pork |
| 3.437 | - | unknown | Beef/Pork |
| 3.446 | - | unknown | Beef/Pork |
| 8.536 | s | L-anserine | Beef/Pork |
| 3.239 | s | L-carnitine | Beef/Pork |
| 3.194-3.203 | s | *O*-acetyl-L-carnitine | Beef/Pork |
| 3.113 | - | unknown | Beef/Pork |
|  |  |  |  |
| 8.275 | s | inosine | Beef/Lamb |
| 8.374 | s | inosine | Beef/Lamb |
| 6.104-6.122 | d | inosine | Beef/Lamb |
| 4.455 | m | inosine | Beef/Lamb |
| 4.284-4.293 | m | inosine | Beef/Lamb |
| 3.905 | m | inosine | Beef/Lamb |
|  |  |  |  |
| 8.230 | s | hypoxanthine | Beef/Lamb |
| 8.248 | s | hypoxanthine |  |
| 3.140 | - | unknown | Beef/Lamb |
|  |  |  |  |
| 3.230-3.239 | s | L-carnitine | Poultry/non-Poultry |
| 8.500 | s | L-carnosine | Poultry/non-Poultry |
| 3.122 | - | unknown | Poultry/non-Poultry |
| 3.221 | m | L-anserine | Poultry/non-Poultry |
| 7.248 | s | L-anserine | Poultry/non-Poultry |
| 3.842 | s | L-anserine | Poultry/non-Poultry |
| 8.536 | s | L-anserine | Poultry/non-Poultry |

**Table S3:** Results of a validation test set of five processed meat products consisting of poultry and pork or beef and pork meat for both classification models (**A**: polar metabolites; **B**: mid-level data fusion). If the *p*-value is ≤ 0.05, the samples do not meet the specified significance level. P, pork; B, beef; Pou, poultry.

|  |  | |  | **A**  **polar metabolites** | | | **B**  **mid-level**  **data fusion** | | |
| --- | --- | --- | --- | --- | --- | --- | --- | --- | --- |
| **n** | declaration | composition | | *p*val  (P) | *p*val (B) | *p*val  (Pou) | *p*val  (P) | *p*val (B) | *p*val  (Pou) |
| **1** | Organic salami (beef and pork) | 50 % beef  50 % pork | | 0.03 | 0.11 | 0.00 | 0.00 | 0.01 | 0.01 |
| **2** | Organic salami (beef and pork) | 75 % pork  25 % beef | | 0.03 | 0.06 | 0.00 | 0.00 | 0.01 | 0.01 |
| **3** | Poultry salami with pork | 53 % poultry  47 % pork | | 0.03 | 0.00 | 0.04 | 0.00 | 0.03 | 0.02 |
| **4** | Delicacy salami (turkey and pork meat) | 53 % poultry  47 % pork | | 0.03 | 0.00 | 0.04 | 0.00 | 0.03 | 0.00 |
| **5** | Poultry salami with pork bacon | 91 % poultry  9 % pork | | 0.00 | 0.00 | 0.15 | 0.00 | 0.00 | 0.03 |
